# Treatment with a neutralizing anti-murine interleukin-17 antibody after the onset of coxsackievirus b3-induced viral myocarditis reduces myocardium inflammation

**DOI:** 10.1186/1743-422X-8-17

**Published:** 2011-01-14

**Authors:** Yang Fan, Wu Weifeng, Yan Yuluan, Kong Qing, Pang Yu, Huang Yanlan

**Affiliations:** 1 Guangxi Cardiovascular Institute, Shuang-Yong Road 6, Nanning, PR China; 2 Department of Cardiology, the First Affiliated Hospital of Guangxi Medical University, Shuang-Yong Road 6, 530021 Nanning, PR China

## Abstract

**Background:**

Recently, some studies indicate that interleukin (IL)-17, known as a T cell (Th17)-derived proinflammatory cytokine, is the major mediator of tissue inflammation in inflammatory and autoimmune diseases. Viral myocarditis (VMC) is a T cell-mediated autoimmune disease, but the role for IL-17 in VMC is not well defined.

**Results:**

Using IL-17 monoclonal antibody (IL-17mAb)-treated VMC mice, we tested the pathogenic role of IL-17 in the development of VMC. VMC mice were treated with monoclonal rat anti-murine IL-17 antibody (anti-IL-17) or rat IgG_2A _isotype control or phosphate-buffered solution 3 days after Coxsackievirus B3 (CVB3) injection. Normal mice without any manipulation were taken as normal control. The survival rates of mice were monitored and heart pathology was examined histologically. IL-17, IL-6, and TNF-α mRNA of the myocardium were assessed by semi-quantitative RT-PCR. Systemic IL-17, IL-6, and TNF-α level were measured by enzyme-linked immunosorbent assay, and local myocardium IL-17 expression was analyzed using immunohistochemical staining. Flow cytometric analysis was used to evaluate the frequencies of Th17 subsets in CD4^+^T cells. Results showed that neutralization of IL-17 with anti-IL-17 can ameliorate clinical symptoms, defer disease course, decrease serum IL-17 level, without declining the IL-17, IL-6 and TNF-α mRNA transcript level and serum IL-6, TNF-α level. The differentiation and proliferation of the Th17 cells were unchanged.

**Conclusions:**

Our data suggest that IL-17 is crucially involved in the pathogenesis of murine VMC, IL-17 inhibition might ameliorate the myocardium inflammation after the onset of VMC.

## Background

Coxsackievirus B3 (CVB3), a member of the Picornaviridae family, is the leading cause of viral myocarditis, which can develop into dilated cardiomyopathy[1,2]. Both the direct viral response and immune-mediated mechanisms have been shown to contribute to the pathogenesis of acute injury and subsequent cardiac remodeling [3,4]. Until now, there is no effective therapy for this disease [5]. Infection of CVB3 in BALB/c murine model can induce myocarditis with a pathological process resembling human disease, thus this model has been widely used for studying both the acute infectious phase and chronic immune phase of human viral myocarditis [6,7]. In past times, a multitude of studies had investigated the role of the Th1 and Th2 mediated cytokine pattern present in animals with VMC. However, it has been demonstrated that IL-23 rather than IL-12 is critical for the initiation of inflammatory and antuimmunity diseases [8,9]. IL-17, a crucial effector cytokine specifically triggered by IL-23, has been shown to be an essential inflammatory mediator in other autoimmune diseases and inflammatory conditions, including VMC [10-15]. Therefore, in the present study, the IL-17 monoclonal antibody (IL-17mAb) was given to VMC mice in order to investigate the therapeutic efficacy of IL-17 neutralization in VMC mouse model.

## Results

### IL-17mAb alleviated the development of myocarditis

**Figure 1:**
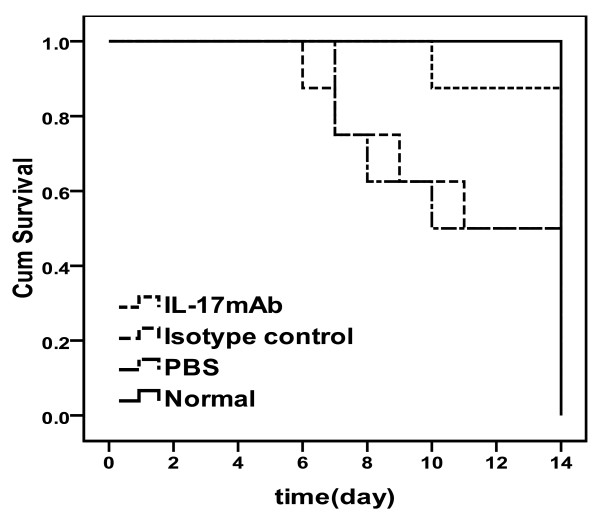
**Effects of anti-IL-17 cytokine therapy on survival rate**. The survival rate of IL-17mAb group mice was significantly improved comparing with the isotype control and PBS groups (*P *< 0.05). No statistical difference was seen between the IL-17mAb and normal mice (*P *> 0.05). Eight mice in normal group, seven mice in IL-17mAb group, four mice in isotype control group and five mice in PBS group survived to 14 days.

Results showed that IL-17mAb alleviate the severity of myocarditis. The survival rate of IL-17mAb group mice were significantly improved comparing with the isotype control and PBS groups [Figure 1]. The number of mice survived to 14 d was 8, 7, 4 and 5 for normal, IL-17mAb, isotype control and PBS groups separately. Statistical differences were seen when comparing the survive rate of anti-IL-17 therapy with that of isotype control or PBS groups (*P *< 0.05), There was no statistical difference of survival rate between isotype control and PBS groups (*P *> 0.05), and no statistical difference was seen between the IL-17mAb and normal mice (*P *> 0.05).

### IL-17mAb alleviated the severity of VMC

**Figure 2:**
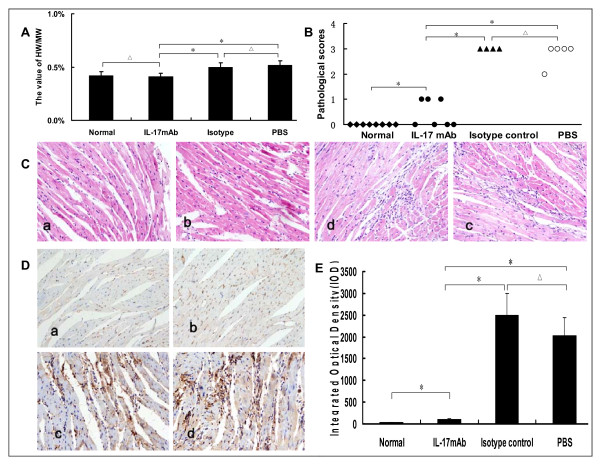
**Evaluation of the severity of VMC**. A, The value of heart weight/body weight (HW/BW) in different groups. The value for each group was 0.42 ± 0.07%, 0.41 ± 0.04%, 0.50 ± 0.05% and 0.52 ± 0.04%, respectively. B, The pathological scores in different groups. Scores for each group were 0.0 ± 0.0, 0.4 ± 0.5, 3.0 ± 0.0 and 2.8 ± 0.4 respectively. Each point represents an individual mouse. C, Representative of histopathological images in heart tissue (H&E, original magnification×400), a: Normal group; b: IL-17mAb group; c: Isotype control group; d: PBS group. D, Representative of IL-17 Immunohistochemistry images in heart tissue (dark brown granules, original magnification×400). a: Normal group; b: IL-17mAb group; c: Isotype control group; d: PBS group. E, Morphometric quantitation of IL-17 protein expression in myocardium. IOD in different groups were 36.04 ± 4.17, 106.41 ± 12.10, 2500.95 ± 65.83 and 2029.63 ± 69.21 respectively. Representative myocardial sections from normal (n = 8), IL-17mAb (n = 7), isotype control (n = 4) and PBS (n = 5) mice. △*P *> 0.05, *P < 0.05. Data are mean ± SD.

The value of HW/BW, pathological scores of heart sections, IOD of IL-17 expression in mice receiving IL-17mAb were lower than those of isotype control and PBS mice (*P *< 0.05), but the pathological scores and IOD of IL-17 expression of IL-17mAb treated mice were a little higher than normal mice (*P *< 0.05). There was no significant difference of the HW/BW, the pathological scores, and IOD between the isotype control and PBS groups (Figure. 2A, B, C, D, E, *P *> 0.05).

### IL-17mAb reduced circulating level of IL-17

**Figure 3:**
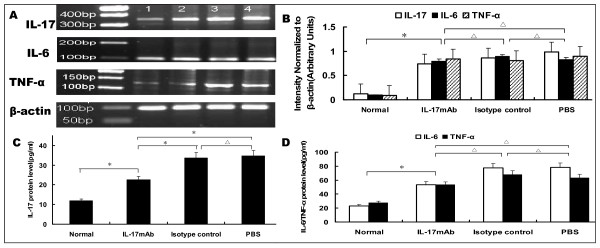
**IL-17, IL-6 and TNF-α mRNA transcription in VMC myocardium and protein level in the serum**. A. Representative images showing semiquantitative RT-PCR for IL-17, IL-6 and TNF-α transcription. 1. Normal group; 2. IL-17mAb group; 3. Isotype control group; 4. PBS group. B, Densitometric quantitation of the PCR bands showed that, among the IL-17mAb, isotype control and PBS groups, the mRNA expression did not differ with each other; C, The levels of serum IL-17 in different groups, measured by ELISA. The IL-17 protein levels were 11.81 ± 2.66, 22.56 ± 3.68, 33.63 ± 5.50, 33.68 ± 6.13 pg/ml in normal, IL-17mAb, Isotype control and PBS group respectively. D, The levels of serum IL-6 and TNF-α in different group, measured by ELISA. The IL-6 levels were 23.15 ± 8.59, 71.28.50 ± 15.80, 77.81 ± 6.54, 78.18 ± 6.26 pg/ml, and the TNF-α levels were 27.80 ± 4.52, 64.24 ± 6.71, 67.89 ± 5.25, 63.45 ± 2.71 pg/ml respectively. Normal group (n = 8), IL17mAb (n = 7), isotype contro(n = 4), and PBS group(n = 5).*P < 0.05, △*P *> 0.05. Data are mean ± SD.

To further determine whether IL-17 was involved in the pathology of VMC, we used RT-PCR to compare the transcriptional levels of IL-17 in heart tissue and the related cytokines IL-6, TNF-α. These cytokines have previously been shown to be involved in myocarditis and may play a role in long-term immunity. Compared with the normal group, the levels of cardiac IL-17, IL-6 and TNF-α mRNA in the IL-17mAb, isotype control and PBS groups were elevated dramatically (*P *< 0.05), but no statistical difference were seen when compared the levels of cardiac IL-17, L-6, and TNF-α mRNA among IL-17mAb, isotype control and PBS groups(*P *> 0.05, Figure 3A, B). The levels of serum IL-17 in the IL-17 mAb, isotype control and PBS groups were higher when compared with those in the normal mice, especially in the isotype control and PBS groups (*P *< 0.05, Figure. 3C). Treating with IL-17mAb reduced the level of total circulating IL-17, which was much lower than those of the isotype control and PBS groups (*P *< 0.05) but still higher than that of normal mice (*P *< 0.05). No significant difference was seen between the isotype control group and PBS group (*P *> 0.05, Figure. 3C). The levels of serum IL-6 and TNF-α in the IL-17mAb, isotype control and PBS groups also increased when compared with those in the normal group (*P *< 0.05), and there were no significant differences among the IL-17 mAb, isotype control and PBS groups (*P *> 0.05, Figure. 3D), which imply that IL-17mAb could not reduce the level of total circulating IL-6 and TNF-α.

### IL-17mAb did not reduce percentages of CD4^+ ^Th17 cells

**Figure 4:**
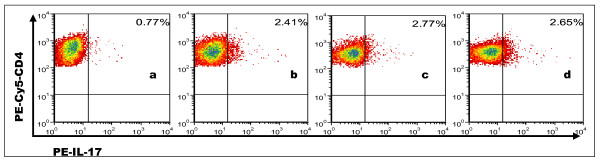
**The percentages of Th17 cells investigated by flow cytometry in each group of spleens**. Th17 subsets were gated with CD4^+ ^IL-17^+^/CD4^+ ^cells. The percentage of positive cells was shown in each panel. Numbers in upper right quadrants represented an average number of each group. a: Normal group; b: IL-17mAb group; c: Isotype control group; d: PBS group.

Compared with the normal group, the percentages of CD4^+ ^Th17 cells in the IL-17 mAb, isotype control and PBS groups increased markedly (*P *< 0.05, Figure 4). Although the percentage of CD4^+^Th17 cells in the IL-17 mAb group trended lower than that of the isotype control and PBS groups, there were no significant differences among them (*P *> 0.05). Th17 frequencies in the normal, IL-17 mAb, isotype control and PBS group were 0.77 ± 0.21%, 2.41 ± 0.57%, 2.77 ± 0.78% and 2.65 ± 0.63%, respectively.

## Discussion

VCM is a T cell-mediated autoimmune disease. The classical theories suggest that Th1/Th2 cytokine balance plays an important role in the pathogenesis of VMC. Th17 cells have been recognized a unique CD4^+ ^T helper-cell (Th) subset, which is characterized by production of IL-17 [16,17]. Emerging data suggest that Th17 cells may play very important roles in inflammatory and autoimmunity diseases, including VCM [10-15]. Demonstration of the role of IL-17 in many inflammatory and autoimmunity conditions further support the concept of IL-17 targeting for treatment. Significantly, recent clinical trials with short duration IL-17 antagonists' therapy in established rheumatoid arthritis (RA) providing the direct evidence in pathological role of IL-17 in RA, and indicating that blockade of IL-17 in human may be a valid therapeutic approach[18]. Therefore, in the present study, the IL-17mAb was given to BALB/c mice after CVB3 infection to investigate whether IL-17 antibody treatment can influence the differentiation and proliferation of Th17 cells after the onset of VMC, thus to be may be a valid therapeutic approach for VMC.

Our results showed that IL-17mAb improved the survival rate of treated VMC mice. Consistently, histological analysis of heart sections revealed that IL-17 mAb attenuate the severity of myocarditis, verified by the decreased HW/BW, a relief of myocardial inflammation, and improved pathological score of heart sections. All the above data indicate that IL-17mAb could rescue mice from myocarditis caused by CVB3 infection, indicating a protective effect of IL-17mAb. To gain insight into the mechanism of action during IL-17 neutralization, Th17 cell frequency, IL-17, IL-6, TNF-α mRNA, serum levels of IL-17 and IL-6 were measured in all animals at the time they were sacrificed. IL-17mAb reduced the circulating level of IL-17, but neither IL-17/IL-6/TNF-α mRNA nor circulating IL-6/TNF-α decreased. TNF-α was secreted primarily by myocytes and macrophages after injury as well as Th1 and Th17 cells [19,20]. Previous studies have demonstrated that cardiac-specific expression of TNF-α result in myocardial inflammation, cardiac hypertrophy, progressive dilatation and increased apoptosis, which leads to heart failure and death [21]. A role for TNF-α in VMC is supported by the recent findings that lack of TNF-a induction is a major factor in female resistance to VMC, exogenous TNF-a also makes normally CVB3 myocarditis-resistant female mice susceptible[22]. IL-6, produced by cells of the innate immune system such as dendritic cells (DCs), monocytes, macrophages, mast cells, B cells, and subsets of activated T cells, including Th17 cells, is clearly a pleiotropic cytokine with multiple effects. As IL-17 can induce the release of many cytokines, such as IL-6, TNF-α and IL-1β, from monocytes, epithelial cells, and fibroblasts et al, when VMC mice were given IL-17 antibody, although circulating IL-17 decreased, the secretion of IL-6 and TNF-α from monocytes et al did not decrease. As an independent subset of T helper cells, Th17 cells were established by the identification of differentiation factors and transcription factors that are unique to Th17 cells. The differentiation factors (TGF-β plus IL-6 or IL-21) [23-25], the growth and stabilization factor (IL-23), and the transcription factors (STAT3, RORγt, and RORα) involve in the development of Th17 cells [26,27]. We have previously demonstrated that Th17 cells differentiate and proliferate from day 7 after CVB3 infection [28], with IL-23, together with other cytokines such as IL-6 and TGF-β expression increased (unpublished observations). In present study, IL-17mAb was gave 3 days after CVB3 infection, in addition with the less but ongoing inflammation, the cytokines proposed to be the differentiation factors for Th17 cells still exist, as a result, there was no influence on the differentiation and proliferation of Th17 cells.

Since there was no complete reduction of the VMC symptoms and the inflammation of myocardium, some causes may elucidate for this. First, as we did not quantify the neutralization of cytokine antibody biological activity, the dose of given IL-17mAb might not be able antagonism with circulating IL-17 in vivo completely. Second, the biological activity, affinity and/or potency of IL-17 antibody in vivo are uncertain. What more, other factors may compensate for the insufficient effect of IL-17. There is a little difference with the previous one, which may be explained by the internal of time and the total amount of antibody given [29].

## Conclusions

IL-17 plays a central role in the development of VMC. Neutralization of IL-17 with a monoclonal antibody starting after onset of VMC result in an attenuated VMC course and ameliorate clinical symptoms. However, several questions remain unset. Since there was no complete reduction of the VMC symptoms, other factors seem to compensate for the temporary deficiency of IL-17 and provide efficient effector cells recruitment, secondary loss of response to IL-17 inhibition may result from the induction of other pathways and replacing the initial IL-17 contribution. Thus, the role of IL-17 inhibition in the treatment of inflammatory conditions remains to be defined.

## Methods

### Mice

Specific pathogen-free male BALB/c mice (6-week-old) were purchased from Shanghai Laboratory Animal Centre, Chinese Academy of Sciences, Shanghai, China (Certificate No.0062353-SCXK (SH) 2007-0005). Ethical approval was obtained from the Guangxi Medical University Animal Ethics Committee before the start of the study. All animals were housed in a pathogen-free mouse room in an experimental animal center (Guangxi Medical University), fed with normal mouse chow and tap water ad libitum.

### CVB3 Titration in Cells

Heart-passaged CVB3 (Nancy strain, from Institute of immunology of Guangxi Medical University) was propagated in Hep-2 cells, cultured in monolayer and stored in a -80°C freezer. The supernatant from infected cell cultures was collected, and viral titers were determined in 96-well plates by the end-point dilution method. Briefly, 10-fold serial dilutions (1:10 to 1:10^-10^) of phosphate-buffered solution (PBS, Solarbio Science & Technology Co, Ltd, Beijing, China) were prepared, and the 50% tissue culture infectious dose (TCID_50_) titer was determined by the cytopathic effects visible after 72 h. The TCID_50 _assay result for Hep-2 cells was 1×10^-7^.

### IL-17 neutralization

Mice were intraperitoneally injection (i.p) with 0.1 ml of PBS containing approximately 100TCID_50 _of the virus. The day when mice were i.p was defined as day 0. Followed by injection, the mice were injected intraperitoneally with 100 μg IL-17 monoclonal antibody (n = 8, R&D Systems, Inc. Minneapolis, MN. MAB421, IL-17mAb group), or 100 μg isotype control immunoglobulin (Ig) G_2A_Ab (n = 8, R&D, Systems, Inc. Minneapolis, MN. MAB006, isotype control group), or PBS (n = 8, PBS group) on day 4, 7 and day 10. In addition, left uninfected and without any intervention mice were assigned as the normal control (n = 8, normal group). All surviving animals were sacrificed on day 14 after CVB3 infection, the values of the heart weight/body weight (HW/BW) were recorded.

### Histopathology

The ventricular tissues of the hearts were cut longitudinally, fixed in 10% phosphate-buffered formalin, embedded in paraffin. The myocardial tissues were cut into 5-μm sections and stained with hematoxylin & eosin to determine the level of inflammation. Stained sections were viewed using light microscopy(Nikon Eclipse E800 Microscope, Kawasaki, Kanagawa, Japan), and graded in a blinded manner by two pathologists based on the following semi-quantitative scale: 0, no inflammatory infiltrates; 1, small foci of inflammatory cells between myocytes or inflammatory cells surrounding individual myocytes; 2, larger foci of 100 inflammatory cells or involving at least 30 myocytes; 3, 10% of a myocardial cross-section involved; and 4, 30% of a myocardial cross-section involved [30].

### Immunohistochemistry

Immunohistochemical staining was performed by the streptavidin-biotin complex method. Rabbit polyclonal antibodies against mouse IL-17 (Santa Cruz Biotechnology, CA) at a 1:100 dilution were used as primary antibodies. The sections were washed and stained using the streptavidin-biotin complex kit (Boster, Wuhan, China) according to the manufacturer's manual. The procedures were as follows: after the sections were rehydrated, endogenous peroxidase activity was blocked with 3% hydrogen peroxide for 10 min at room temperature. After washing with distilled water, the sections were first incubated in 5% bovine serum albumin for 20 minutes at room temperature and then in the primary antibody for 24 h at 4°C. Next, the sections were washed with PBS and then incubated with the streptavidin-biotin complex for 20 minutes. Finally, the sections were developed with 3,3-diaminobenzidine (Boster, Wuhan, China) and observed under a light microscope (Eclipse E800, Nikon, Japan). Non-immune goat serum was used instead of primary antibody as a control. IL-17 deposition in the cytoplasm and cytomembrane were categorized semi-quantitatively according to the extent and intensity of staining using Image Pro Plus Version 6.0 (Media Cybernetics, Bethesda, MD). Two pathology experts randomly selected 5 fields from each slice for blinded scoring and analysis by integrated optical density (IOD).

### Lymphocyte preparation

Spleens from the four groups of mice were harvested aseptically. After mincing, the cell suspension was pipetted rapidly with a sterile Pasteur pipette into 3 ml of RPMI 1640(Gibco, USA), filtered through nylon mesh to eliminate debris, and centrifuged at 1000 rpm for 5 min. The cells were washed twice with RPMI 1640. The lymphocyte fractions of these samples were obtained by Ficoll Paque (Solarbio Science &Technology) gradient centrifugation, the suspension was layered carefully over 3 ml of Ficoll Paque and centrifuged at 2000 rpm for 15 min. Lymphocytes were maintained in a 24-well flat-bottom tissue culture plate with RPMI 1640 supplemented with 10% fetal calf serum (Gibco, USA) at 37°C in a humidified atmosphere with 5% CO_2_.

### Intracellular cytokine flow cytometry

Cytokine-producing cells were determined by intracellular staining using phycoerythrin-conjugated anti-mouse IL-17 (PE-IL-17) and phycoerythrin cyanine-5-conjugated anti-mouse CD4 (PE-Cy5-CD4). Briefly, cells were stimulated with phorbol myristate acetate (25 ng/ml, Sigma-Aldrich), ionomycin(1 μg/ml, Sigma-Aldrich), and GolgiPlug (1 μl/10^6 ^cells, BD Biosciences) for 4 hours. Cells were fixed in 4% paraformaldehyde, permeabilized with 0.1% saponin, stained with fluorescent antibodies against IL-17 and CD4, and analyzed on a FACSCalibur flow cytometer (BD Biosciences). CellQuest software (BD Biosciences) was used for data acquisition.

### Semi-quantitative RT-PCR detection of IL-17, IL-6, TNF-α mRNA

Total mRNA was extracted from homogenized heart tissues by using of the TRIZOL Reagent^® ^(Invitrogen, USA), and then used to synthesize cDNA with an RT Kit (Ferma, USA). Reverse-transcription PCR (RT-PCR) was performed with first-strand cDNA synthesized with 1 μg of total RNA and oligo d(T)18 primers according to the manufacturer's instructions. The primers for the RT-PCR assays for IL-17A, IL-6 and TNF-α were designed by Primer Premier 5.0 (Table 1). Mouse β-actin, a reference gene, was used to normalize each sample and each gene. Prepared cDNA was used for PCR amplification with the above primers under the following conditions: pre-heating at 94°C for 3 min, denaturing at 94°C for 30 sec, annealing at at 64.9°C (IL-17) or 62°C (IL-6) or 62.5°C (TNF-α) for 30 sec, and extension at 72°C for 60 sec. The reaction repeated for 35 cycles followed by incubation at 72°C for 10 min. PCR products were analyzed by electrophoresis on a 2% agarose gel containing 0.5 mg/ml ethidium bromide. The resulting bands were observed and photographed under ultraviolet light, and then measured using the Digital Gel Imaging Analyst (Nikon 990-Doc 1000, USA). Density was determined for each sample PCR product, including the positive control. Background density was subtracted from each band and the relative values of IL-17, IL-6 and TNF-α mRNA were calculated using β-actin mRNA as a standard. PCR products was sequenced by Songon Biotech Co., Ltd (Shanghai, China), and blasted in the NCBI Blast bank.

**Table 1:** Sequences of primers for RT-PCR

Molecule	Sequence (5'-3')	length
IL-6[GenBank:16193]	sense: 5'CACAGAAGGAGTGGCTAAGGACCA3' antisense: 5'ACGCACTAGGTTTGCCGAGTAGA3'	103 bp

IL-17[GenBank:16171]	sense: 5'GTCAATGCGGAGGGAAAG3' antisense: 5'CACGAAGCAGTTTGGGAC 3'	349 bp

TNF-α GenBank:21926]	sense: 5'CACTGGAGCCTCGAATGTC3' antisense: 5'CAGGGAAGAATCTGGAAAGGT3'	128 bp

β-actin [GenBank:11461]	sense: 5'CCAGCCTTCCTTCTTGGGTAT3' antisense: 5'TTGGCATAGAGGTCTTTACGG 3'	102 bp

### Cytokine assay

Blood were collected via retro-orbital bleeding and serum was separated. The amounts of IL-17 in the blood plasma were detected using the Quantikine Mouse IL-17 Immunoassay (R&D Systems, Minneapolis, MN, USA) and the amounts of IL-6, TNF-α were detected by Quantikine Mouse IL-6, TNF-α Immunoassay (Shanghai ExCell Biology Inc. China). The minimum detectable doses of IL-17, IL-6 and TNF-α were 5, 15.6 and 15.6 pg/ml respectively. No cross-reactivity was observed in detection. All samples were measured in triplicate.

### Statistical analysis

All data were expressed as mean ± SD. One-way ANOVA and the *q *tests were used for comparison among four groups. All data were analyzed with SPSS 16.0 for Windows. A significant difference was defined as *P *< 0.05.

## Competing interests

The authors declare that they have no competing interests.

## Authors' contributions

YF participated in data collection, coordinated the study, performed the statistical analysis and interpretation of data, prepared the draft of manuscript and reviewed it. WW conceived of the study and designed it, coordinated the study and reviewed it. YY, KQ, PY carried out data collection. HY participated in design of the study and reviewed the manuscript. All the authors have read and approved the final manuscript.
